# Genome-wide search identifies *Ccnd2 *as a direct transcriptional target of Elf5 in mouse mammary gland

**DOI:** 10.1186/1471-2199-11-68

**Published:** 2010-09-10

**Authors:** Rosalba Escamilla-Hernandez, Rumela Chakrabarti, Rose-Anne Romano, Kirsten Smalley, Qianqian Zhu, William Lai, Marc S Halfon, Michael J Buck, Satrajit Sinha

**Affiliations:** 1 Department of Biochemistry, State University of New York at Buffalo, Center for Excellence in Bioinformatics and Life Sciences, Buffalo, NY 14203, USA

## Abstract

**Background:**

The ETS transcription factor Elf5 (also known as ESE-2) is highly expressed in the mammary gland and plays an important role in its development and differentiation. Indeed studies in mice have illustrated an essential role for Elf5 in directing alveologenesis during pregnancy. Although the molecular mechanisms that underlie the developmental block in Elf5 null mammary glands are beginning to be unraveled, this investigation has been hampered by limited information about the identity of Elf5-target genes. To address this shortcoming, in this study we have performed ChIP-cloning experiments to identify the specific genomic segments that are occupied by Elf5 in pregnant mouse mammary glands.

**Results:**

Sequencing and genomic localization of *cis*-regulatory regions bound by Elf5 *in vivo *has identified several potential target genes covering broad functional categories. A subset of these target genes demonstrates higher expression levels in Elf5-null mammary glands suggesting a repressive functional role for this transcription factor. Here we focus on one putative target of Elf5, the *Ccnd2 *gene that appeared in our screen. We identify a novel Elf5-binding segment upstream of the *Ccnd2 *gene and demonstrate that Elf5 can transcriptionally repress Ccnd2 by directly binding to the proximal promoter region. Finally, using Elf5-null mammary epithelial cells and mammary glands, we show that loss of Elf5 *in vivo *leads to up regulation of Ccnd2 and an altered expression pattern in luminal cells.

**Conclusions:**

Identification of Elf5-targets is an essential first step in elucidating the transcriptional landscape that is shaped by this important regulator. Our studies offer new toolbox in examining the biological role of Elf5 in mammary gland development and differentiation.

## Background

Ets transcription factors are highly conserved proteins that have a unique 85 amino acid DNA-binding domain. Ets proteins activate or repress the expression of a myriad of genes that are involved in various biological processes, including cellular proliferation, apoptosis, differentiation, and transformation[1]. Typically, Ets proteins directly bind to regulatory elements such as promoters and enhancers that contain a GGAA/T core sequence motif thereby regulating target gene expression. This protein family consists of 25-30 members in mammals, which are broadly expressed in a variety of tissues and their relative expression differs according to cell type. This poses a challenging task of determining which of these Ets proteins are biologically active in a given cellular environment and to link a specific Ets protein to its target.

The mammary epithelium and cell lines derived from mammary tissues and tumors express a number of Ets factors[2-4]. The critical function of some of these Ets factors in mammary gland development, differentiation and tumorigenesis is dramatically reflected in the phenotypes observed in transgenic and knockout mouse studies[3]. One such Ets factor is Elf5 (also called ESE-2), which is highly restricted to tissues and organs rich in glandular or secretory epithelial cells including the mammary luminal epithelium[5,6]. The first hint as to the functional importance of Elf5 in mammary gland development came from studies showing that Elf5 heterozygous female mice display impaired mammary alveolar morphogenesis[7]. However, the fact that Elf5-null mice die very early during embryogenesis due to developmental defects in the formation of extraembryonic ectoderm limited further studies[8]. To overcome this, we and others have investigated the effects of the complete loss of Elf5 using tissue-specific knockout models and mammary transplants[9,10]. Mammary glands that are deficient in Elf5 completely fail to initiate alveologenesis during pregnancy and retain characteristics of virgin ductal epithelial cells. Furthermore, Elf5-null mammary glands accumulate CD61+ luminal progenitor cells supporting a critical role for Elf5 in specifying the differentiation of mammary epithelial progenitors to establish secretory alveolar lineage[9]. Our studies have also shown that Elf5 transcriptionally regulates the expression of key mediators of the Prolactin/Jak2/Stat5 signaling pathway, and alterations in this pathway might be partly responsible for the Elf5-null mammary gland phenotype[10]. These studies have unearthed a wealth of information about the biological role of Elf5 in mammary gland development and established Elf5 as a critical transcription factor that dictates cell fate and lineage choices.

While the importance of Elf5 in normal mammary gland development is firmly established, whether it acts as a tumor suppressor or an oncogene in breast tumorigenesis remains to be determined. Interestingly, ELF5 is localized to human chromosome 11p13-15, a region of the genome, which undergoes loss of heterozygosity (LOH) in many types of cancer, including ductal carcinoma of the breast[5]. Preliminary studies have demonstrated that loss of Elf5 is frequently found in human mammary carcinoma cells and Elf5 mRNA expression also is lost in a number of breast cancers compared to adjacent normal tissues[5,11]. These observations are in agreement with a recent study on MMTV-*Wnt-1 *murine breast tumors, which showed that Elf5 expression was significantly diminished in the tumorigenic compartment[12]. However these results are in contrast to expression analysis studies of breast cancer by other laboratories, which have suggested increased Elf5 expression in breast cancer[2,4]. Notwithstanding the lack of a clear-cut role of Elf5 in tumor development, it is safe to posit that this transcription factor is an important mediator of various facets of mammary gland biology and warrants further experimental studies. Identification of the repertoire of its target genes is one such critical step in better understanding the molecular mechanisms underlying Elf5 function.

Our biochemical studies have demonstrated that the Ets domain of Elf5 acts as a transcriptional repressor, whereas the N-terminal Pointed domain can function as a transcriptional activator[13]. These observations fit well with studies on some putative Elf5-target promoters such as PSA, SPRR2A, Keratin 8, and WAP promoters, which have shown that Elf5 can activate or repress transcription in a context-dependent manner[9,10,14]. However, a global analysis of Elf5-binding events is lacking, and our current knowledge of what are bona-fide Elf5-targets is extrapolated from a limited number of examples that have been obtained from in vitro binding studies and reporter assays in keratinocytes. To overcome this shortcoming, here we have applied a chromatin immunoprecipitation (ChIP)-cloning strategy with validated ChIP-grade anti-Elf5 antibodies to identify genetic loci bound by Elf5 in mammary epithelium. Our studies have identified numerous physiologically relevant downstream targets of Elf5 including several of known significance in the development and function of mammary glands. As a proof of principle to demonstrate the validity of our approach and to further elucidate the role of Elf5 in mammary glands, we have focused our studies on Ccnd2 (cyclin D2). We show that Elf5 binds to a conserved site within an upstream regulatory element as well as the proximal promoter of the *Ccnd2 *gene and that the expression level of cyclin D2 is upregulated by the loss of Elf5 in mammary epithelial cells in culture as well as in Elf5 deficient mammary glands. Collectively our ChIP approach has identified numerous mouse target genes of Elf5 and has offered insight into the regulatory pathways controlled by Elf5 during mammary gland morphogenesis and cancer.

## Results and Discussion

### Assessing the efficacy of Elf5-antibodies for chromatin immunoprecipitation

**Figure 1:**
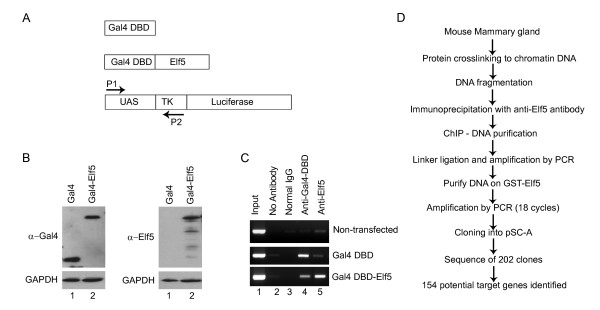
**Assessment of anti-Elf5 antibody for ChIP**. The Elf5 antibody was tested for ChIP using the human cell line HEK293 UAS-TK-Luc, which contains a stably integrated luciferase reporter gene under the control of UAS (containing Gal4 binding sites) and the TK promoter. **A**. *Schematic representation of the plasmids and primers utilized in the experiment*. **B**. *Expression of the Gal4 proteins assessed by anti-Gal4 and anti-Elf5 antibodies*. HEK293 UAS-TK-Luc cells were transfected with plasmids that express Gal4 DNA-binding domain (Gal4 DBD) or Gal4 DBD-Elf5. **C**. *ChIP assays performed with anti-Gal4 DBD or with anti-Elf5 antibodies*. Immunoprecipitated DNA was analyzed using P1 and P2 primers. As a positive control, an aliquot (1%) of chromatin complex before immune isolation was used as input for PCR. Nonspecific binding was judged using rabbit IgG or no antibody. **D**. *Schematic overview of the ChIP protocol used for the cloning of Elf5-putative target genes*.

Although the commonly used commercial Elf5 antibody (N-20) shows robust activity in detecting the endogenous protein by both western blot and immunostaining and has been used sporadically for ChIP experiments, its efficiency in immunoprecipitating endogenous Elf5 has not been experimentally determined. Hence, we took advantage of a recently described Gal4-based cell culture system to test the efficacy of anti-Elf5 antibodies[15]. HEK293 UAS-TK-Luc is a human cell line with a Gal4-responsive luciferase gene integrated into the genome. Our goal was to test if the N-20 antibodies are capable of recognizing its specific epitopes under harsh conditions, those typically associated with ChIP experiments such as formaldehyde crosslinking and stringent washing. This also allowed us to assess how well the N-20 antibodies fare, when compared to ChIP-grade antibodies against another DNA-binding protein such as Gal4. For this purpose, we transfected the HEK293 UAS-TK-Luc cell line with plasmids expressing either the Gal4 DNA binding domain or a chimeric protein consisting of Gal4 DNA binding fused to Elf5 and confirmed the expression of the proteins by western blot analysis with anti-Gal4 and anti-Elf5 antibodies (Fig 1A and 1B). The transfected cells were then subjected to ChIP using antibodies against Gal4 and Elf5. When PCR was performed with ChIPed DNA for the Gal4-responsive UAS region, as expected, a significant enrichment was observed specifically with anti-Gal4 antibodies in cells transfected with plasmids encoding Gal4 DNA binding (Fig 1C). On the other hand, in cells transfected with Gal4 DBD-Elf5, enrichment was obtained when using both the Gal4 and Elf5 antibodies. This enrichment was specific, since control lanes (no antibodies or IgG) did not show any PCR products and only background levels of products were obtained in untransfected cells. Interestingly, under these conditions, the Elf5 antibodies performed as well as, if not better than the Gal4 antibodies, confirming its usefulness for ChIP experiments to detect genomic targets. One potential caveat however is the fact that this experiment was performed on Gal4-Elf5 fusion protein, and the presence of Gal4 DNA binding domain might influence the overall conformation and accessibility of the epitope. Thus it is conceivable that the N-20 antibodies may be more (or less) competent and specific in immunoprecipitating endogenous Elf5.

### Chromatin immunoprecipitation of mammary epithelium with anti-Elf5 antibodies and cloning of Elf5-bound genomic segments

Having established that the N-20 anti-Elf5 antibodies are well suited for ChIP experiments, we next decided to identify the *in vivo *target genes of Elf5 in mouse mammary glands obtained from 17.5 days of pregnancy. Importantly, Elf5 is highly expressed during this stage of alveolar maturation and knockout studies have clearly demonstrated an indispensable role for this transcription factor in alveologenesis. Using the N-20 antibodies, we prepared a library of chromatin-DNA immunoprecipitated from mammary glands. To overcome the technical challenge associated with limiting amounts of DNA obtained during the ChIP procedure, we utilized a ligation-mediated PCR technique, a method successfully used by our laboratory in prior studies (Fig 1D)[16]. In addition, to reduce non-specific DNA contamination, we purified the PCR-amplified fragments by incubation with agarose beads containing GST-Elf5 protein. This enrichment procedure allowed us to select for DNA fragments that are more likely to contain DNA binding-sites for Elf5. Using this strategy, we isolated and sequenced 202 clones. Of the 202 sequenced clones, we found that there were 18 duplicate sequences, and 1 could not be mapped to a specific location in the mouse genome. This resulted in a final 183 unique mouse clones, which were analyzed by mapping them to the mouse genome database by using a variety of search programs including the University of California Santa Cruz genome browser, ENSEMBL, or the BLAST program at NCBI. This allowed us to determine the location of the Elf5-immunoprecipitated DNA fragments in relation to known or predicted genes.

Our study revealed that 154 DNA fragments out of the 183 unique clones immunoprecipitated by anti-Elf5 antibodies were embedded within or located near known, annotated, or predicted genes. We chose to assign the DNA fragments to a specific gene if the sequence matched to the intragenic region or a segment within 100 kb upstream or downstream. The 154 gene-associated Elf5-binding fragments identified by this approach are listed in Table 1 (see Additional File 1) with their genomic coordinates. The remaining 29 fragments that did not map to genomic regions close to any gene may represent distal enhancers involved in regulating gene expression from distances significantly farther than 100 kb, a characteristic found in some enhancers. Alternatively, these segments may denote non-annotated regions of the genome that do not encode for conventional genes but are sites for miRNA and similar elements. Some of these elements may also represent experimental artifacts resulting from non-specific DNA-binding of Elf5 to certain chromatin regions that are captured during formaldehyde cross-linking or contaminating DNA obtained during the immunoprecipitation or PCR enrichment steps.

### Characteristic Features of the Elf5 target regions

**Figure 2:**
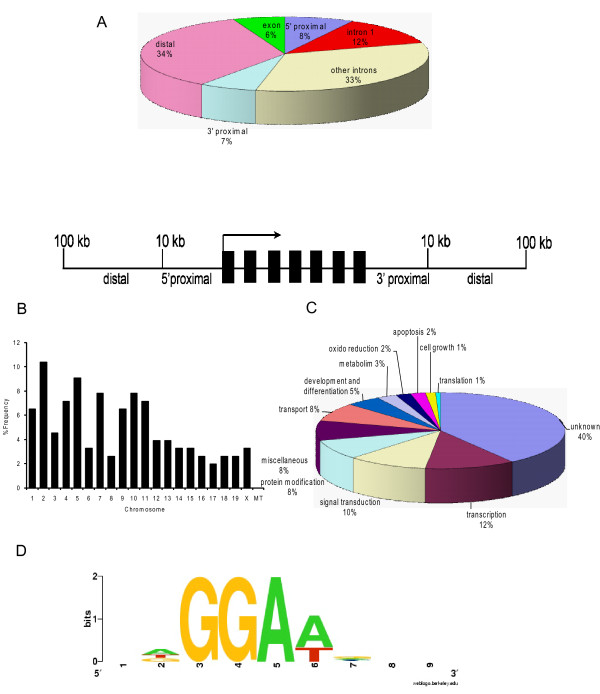
**Localization and functional categories of putative Elf5 target genes**. **A**. *Distribution of Elf5-ChiPed DNA relative to putative target genes*. The schematic below illustrates the assigned locations relative to the gene; the arrow indicates the transcription start site and the black boxes represent exons. **B**. *Chromosomal localization of Elf5-ChIPed DNAs*. **C**. *Distribution of functional categories of putative Elf5 target genes based on gene ontology*. **D**. *A weblogo of Elf5 DNA-binding consensus site *The weblogo was generated based on the Patser analysis of putative Elf5-binding sites recovered from the genomic segments occupied by Elf5.

Examination of the 154 Elf5 occupied target fragments revealed several interesting findings. Forty five percent of the Elf5-ChIP fragments were located within an intron of known or predicted genes, with a large proportion of them in the first intron (Fig 2A). A majority of the DNA fragments chromatin immunoprecipitated by Elf5 was located within a region spanning 100-kb upstream or downstream of candidate target genes. This observation is in agreement with many other transcription factors such as Gata-1, Foxa2, TCF4 for which genomic binding sites have been deciphered on a large scale and reflects the growing evidence for the presence of intragenic and distant extragenic *cis*-regulatory regions for transcriptional control[17-20]. Although several sites were located less than 10-kb upstream of a transcription start site (8%), only a small number mapped to the promoter proximal regions. This may reflect the propensity of Elf5 to act primarily through distal enhancers or simply signify under-representation of promoter regions since they are not well defined and properly annotated for many mouse genes.

Interestingly, some Elf5-ChIPed DNA fragments were located in between two genes. In these cases, although we have only chosen the nearest located gene as a potential Elf5-regulatable gene for subsequent analysis, it is possible the distally located gene may be the actual Elf5-target. For example, one of the immunoprecipitated DNA fragments mapped to a region that is upstream of the Elf5 gene itself suggesting this region could function as an enhancer for Elf5. This is of particular interest given the propensity of many transcription factors to auto-regulate themselves through DNA-binding elements located in their regulatory regions. However, the Elf5 gene lies close to a family member Ehf (also called ESE-3) in a head-to-tail orientation. Due to this close proximity, the Elf5-ChIPed fragment maps to a region ~50 kb away from Elf5 and ~66 Kb from Ehf. In view of the overlapping expression pattern and similar biological role of Elf5 and Ehf in epithelial development, it is quite possible that the Elf5-response element located in the intergenic region may coordinately regulate the expression of both these genes.

Elf5-target sites are broadly located on all mouse chromosomes, suggesting a broad and unbiased distribution across the mouse genome (Fig 2B). Functional classification of these potential targets based on Gene Ontology categorization revealed that these are widely distributed among a wide variety of categories including transcriptional regulation, signaling cascades and metabolism (Fig 2C). The diverse nature of the targets identified in our study reinforces the notion that Elf5 plays a role in complex biological pathways that affect a wide variety of cellular processes. Given the proposed role of Elf5 in regulating the gene expression of milk proteins during alveologenesis, we were surprised that no such gene was found in our ChIP data. Since by some estimates, transcription factors are thought to bind to ~thousands of genomic sites, the absence of any milk protein genes in our Elf5 target list could be due to the small sample size.

We next searched ChIP-identified sequences of the 154 identified targets to find DNA-binding motifs that may be indicative of Elf5 binding sites. Previous studies have attempted to define the consensus Elf5 DNA binding site based on gelshift binding assays and in vitro selection experiments. Although these studies have revealed slightly different consensus sites for Elf5 such as 5'-(A/C)GGAA(A/G)(G/T)(A/G)NNC-3' [14] and 5'-ANCAGGAAGTAN-3' [6] and 5'-(A/C)GGAA(A/G)(G/T)(A/G)NNC-3' [13] - they all contain the invariant GGAA core sequence, but differing flanking sequence. Hence, we searched the 154 Elf5-ChIPed segments in a biased manner using the GGAA position weight matrix (PWM) as a query for the pattern-recognition program Patser. This analysis showed that the GGAA core motif is highly enriched within ChIP-identified Elf5 target regions. Indeed, at least one GGAA motif was identified in all ChIP-cloned fragments, whereas several DNA segments with multiple GGAA elements were identified by the Patser program with e-value cutoff -5. These numbers were lower when a more stringent e-value cut off of -6 was used, however the trend remained the same. The consensus DNA-binding site for Elf5 based on the ChIPed genomic sequence is quite similar to that previously described as shown in the weblogo generated from our current data (Fig 2D). The fact that many of the potential Elf5-binding sites identified by ChIP were highly conserved support the notion that these sites are likely to be functionally relevant (data not shown).

### Confirmation of a subset of Elf5 target genes by independent ChIP assays and their expression levels in the absence of Elf5

**Figure 3:**
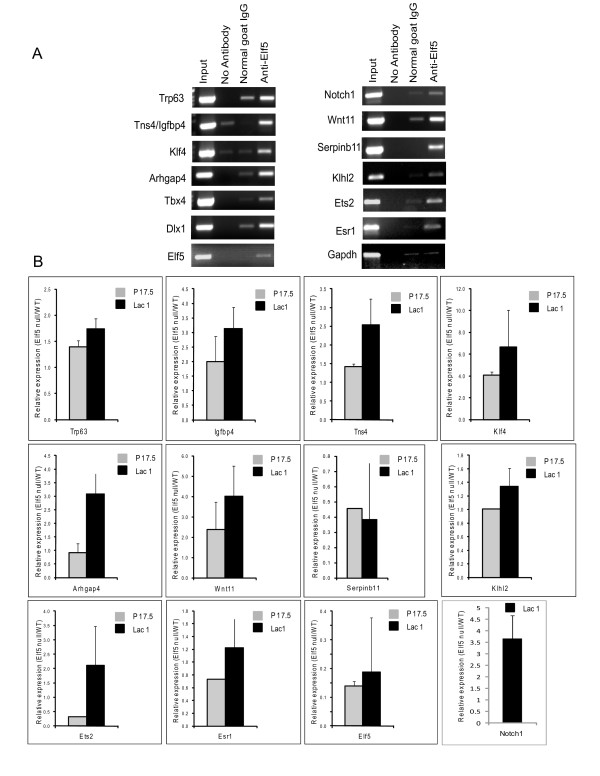
**Verification of putative Elf5 target DNA by independent ChIP and examination of their expression levels in Elf5 null-mouse mammary glands**. **A**. *Independent ChIP assay to demonstrate Elf5 occupancy*. Mouse mammary gland at pregnancy day 17.5 were immunoprecipitated with anti-Elf5 antibodies and analyzed by PCR using specific primers that amplified genomic DNA segments located close to putative Elf5 target genes and Gapdh as negative control. As positive control of PCR, an aliquot (1%) of chromatin complex before immune isolation was used as input. Nonspecific binding was assessed using goat IgG or no antibody. **B**. *Realtime RT-PCR analysis of Elf5 target genes in WT and Elf5-null mammary gland*. Total RNA from wild type (WT) and K14-Cre/Elf5^f/f ^(Elf5 null) mouse mammary glands at pregnancy day 17.5 and lactation day 1 were analyzed for relative expression of mRNA levels of putative Elf5 target genes by real time RT-PCR. Data shown is from at least two independent experiments.

To facilitate further studies of the potential Elf5 targets, we selected thirteen genomic loci identified by ChIP. These DNA fragments immunoprecipitated by Elf5 were chosen to represent the wide range of distinct characteristics such as potential function of the target genes, different locations relative to the target genes (5', 3' or intragenic), and whether the Elf5-binding segments were close to one or more target genes (Additional File 2, Fig S1). However given the importance of Elf5 to mammary gland development and differentiation, we focused on a few genes that were transcription factors (such as *Trp63, ESR1, Ets2*) or key members of signaling pathways (such as *Notch1 and Wnt11*) that are relevant to mammary gland biology. For these experiments, cross-linked chromatin from mouse mammary glands was immunoprecipitated in two independent experiments with anti-Elf5 antibodies and the co-precipitation of the Elf5-response elements was ascertained by PCR with primers that amplify the chosen thirteen fragments. As a negative control we utilized a genomic segment corresponding to a segment of the *Gapdh *gene. As shown in Fig 3, after immunoprecipitation of cross-linked chromatin we found that there was specific enrichment of all potential Elf5-response elements, but not Gapdh with antibodies against Elf5 compared to IgG or no antibody control. Although for most of the genomic fragments, the extent of specific amplification that was observed was robust, in a few cases the PCR product was only marginally stronger than the control suggesting that there is a great deal of variability in the extent of recruitment of Elf5 to these sites. This might reflect the strength and number of Elf5-binding sites, presence of additional transcription factor binding to these regions and/or specific regulatory events that are dictated by the differentiation state of the mammary epithelial cells.

Next we performed real time RT-PCR experiments to test if genes associated with the thirteen genomic loci were indeed expressed in mammary glands and if their expression levels were altered in the absence of Elf5. We have previously shown that mammary epithelium-specific conditional deletion of Elf5 leads to a complete block in alveologenesis during lactation. Given the strong expression of Elf5 in mammary glands at 17.5 day of pregnancy and the dramatic phenotype observed in lactating day 1, these two time frames were chosen for further studies. We isolated RNA from mammary glands from K14-Cre/Elf5^f/f ^animals and their WT littermate controls (K14-Cre/Elf5^+/+^). The real time PCR data revealed that majority of the genes showed significant differences in their level of expression in Elf5-null mammary glands compared to wild type controls (Fig 3B). Interestingly, of the several candidate genes chosen, two of them, *Dlx1 and Tbx4 *showed no detectable expression in mammary glands under our experimental conditions. It is possible that the Elf5-ChIPed fragment located close to the *Dlx1 *gene might alternatively be involved in regulating the *Metapl1 *gene, situated on the 3' end (Fig S1). On the other hand, the complete lack of expression of Tbx4 in mammary gland is more puzzling and raises the possibility that the corresponding genomic fragment obtained in our screen is perhaps non-functional, representing either fortuitous binding or an experimental artifact. One interesting aspect of these studies is that majority of the genes that were examined showed up regulation in the absence of Elf5. This suggests that Elf5 may act broadly as a transcriptional repressor, a property that we had observed in our previous biochemical experiments[13]. Another aspect that caught our attention is that many of the potential Elf5 target genes are myoepithelial/basal restricted (Trp63 for example). This might reflect a propensity for Elf5 to suppress myoepithelial gene expression and foster a luminal cell fate.

### Identification of *Ccnd2 *as a direct Elf5 target

**Figure 4:**
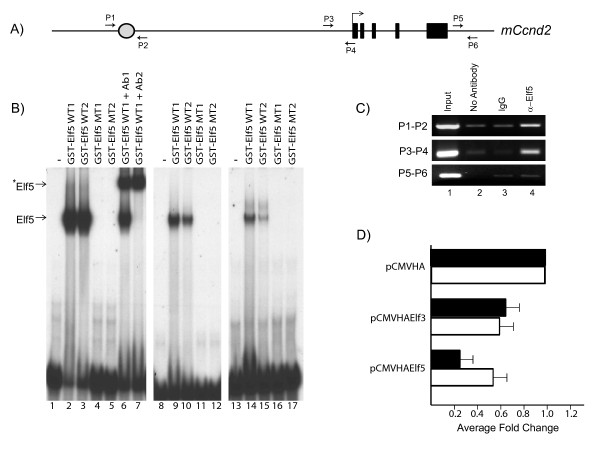
**Binding of Elf5 to regulatory regions of the *CCND2 *gene**. **A**. *A schematic map of the cyclinD2 gene showing the genomic structure and the location of the Elf5-ChIPed segment in the 5' upstream region (circle)*. The primer sets used for ChIP experiments are shown. **B**. *Gelshift experiments to demonstrate binding of recombinant Elf5 to cyclinD2 regulatory regions*. The Elf5-DNA complex is supershifted (*) with the addition of two different antibodies against Elf5, Ab1 (N-20) and Ab2 (generated in the laboratory). Lanes 1-7 represent data with the oligonucleotide corresponding to the cyclinD2 promoter, whereas lanes 8-12 and 13-17 correspond to two segments of the upstream region that contain Elf5-binding sites. **C**. *In vivo occupancy of Elf5 on mouse Ccnd2 gene*. ChIP was performed on mouse mammary glands using antibodies recognizing Elf5 as well as a nonspecific IgG as indicated. Input represents PCR amplification of 1% of the genomic DNA. Primer pairs P1-P2 and P3-4 correspond to the upstream and the promoter region respectively. P5-P6 corresponds to 3' region of *Ccnd2 *gene and serves as a negative control. Results shown are a representative of two independent experiments. **D**. *The cyclin D2 promoter is repressed by Elf5 in reporter gene assays in mammary epithelial cells*. The wildtype (black) or mutant (white) cyclinD2-Luc plasmid was co-transfected with expression plasmids encoding Elf5 or Elf3 into MCF-7 cells. Luciferase values were determined and normalized against β-galactosidase values. The corrected luciferase values for cells transfected with empty vector pCMV-HA were set at 1.

Having shown that our strategy for finding Elf5-targets was successful, we decided to focus on one putative target gene *Ccnd2 *(cyclin D2) for further detailed examination. This was prompted by several observations including a prior study that demonstrated that transgenic mice expressing high levels of cyclin D2 under the MMTV promoter exhibit a lack of alveologenesis and a failure to lactate - a phenotype that mimics that of the Elf5 knockouts[21]. Furthermore, the Elf5 ChIPed fragment corresponded to an evolutionarily conserved DNA segment ~60 Kb upstream of the mouse *Ccnd2 *gene suggesting that this region was likely to play a functional role (see Additional File 3, Fig S2 and Fig 4A). The upstream segment contained two potential Elf5-binding sites. We reasoned that if Elf5 directly regulated the expression of *Ccnd2*, it was likely to also interact with the promoter region. A careful examination of the proximal promoter sequence revealed a consensus Elf5-binding site at -65 to -60 upstream of the transcriptional start site (Additional File 4, Fig S3).

We therefore probed if these potential Elf5-binding sites were directly capable of interacting with Elf5. For this purpose, full-length Elf5 protein was expressed in *E. coli *as a GST-fusion protein. In addition, we also generated Elf5 GST-fusion proteins containing specific mutations in amino acids R219 and K216 located in the DNA-binding domain. We reasoned that based on sequence conservation of the Ets domain, these two amino acids are likely to be critical mediators of Elf5-DNA binding. For example, in case of the ETS transcription factor, PDEF, the Ets domain makes a number of contacts with its DNA substrate[22]. One site of major interaction with bases occur at the conserved arginine residue R307, which make key hydrogen bonds with the GGA core, whereas a highly conserved lysine, K304, is thought to be involved in the tethering of DNA along with other residues to properly orient the DNA molecule. These two residues of PDEF are the counterparts of R219 and K216 in Elf5 and hence likely to be important for DNA-binding. The wildtype and mutant GST-Elf5 proteins were purified to reasonable homogeneity (Additional File 5, Fig S4) and tested for their ability to bind to oligonucleotides containing Elf5 consensus DNA-binding sequences by gelshift experiment. As expected, while wildtype GST-Elf5 protein strongly bound to DNA, the mutants completely failed (data not shown).

We next generated oligonucleotide probes corresponding to the three Elf5-consensus sequences. These probes were labeled to approximately the same level of specific activity and tested by gelshift assays with GST-Elf5 and GST Elf5 mutant proteins. As shown in Fig. 4B, GST-Elf5 showed strong binding to the promoter sequences of cyclin D2 whereas both mutants did not show any detectable binding. The DNA-protein complex could be supershifted with two different antibodies against Elf5 confirming the specificity of the complex (left panel). In a similar fashion, oligonucleotides containing two distinct Elf5-consensus sites embedded within the cyclin D2 upstream element were also capable of forming complexes specifically with GST-Elf5 but not with either of the mutants (middle and right panel). Interestingly, the DNA-protein complex with both the oligonucleotides of the upstream element was relatively weaker as compared to the promoter region. This suggested that the promoter sequence likely corresponded to a high-affinity Elf5-binding site and reaffirmed the notion that sequences flanking the core GGAA motif significantly influence binding activity as demonstrated before.

To clearly demonstrate that *Ccnd2 *is a direct target gene of Elf5 we performed an independent ChIP assay (Fig. 4C). We used anti-Elf5 antibodies to immunoprecipitate crosslinked chromatin from mouse mammary tissue. We designed a set of primers (P1 and P2) to amplify the putative cyclinD2 upstream element identified in our screen and the proximal promoter region (P3-P4). As a control, we designed a set of primers (P5-P6) that amplify a random region of genomic segment 3' of the *Ccnd2 *gene that did not show any sequence conservation. As shown by PCR data in Fig. 4C, specific enrichment of the cyclin D2 5' upstream and promoter region, but not the 3' downstream element was observed after immunoprecipitation with antibodies against Elf5 as compared to the IgG or no antibody control. This suggested that *in vivo *Elf5 physically occupies the regulatory elements of the *Ccnd2 *gene and given the presence of Elf5-binding sites, we posit that this is a direct interaction.

Having demonstrated that Elf5 can directly bind to the cis elements of the *Ccnd2 *gene, our next goal was to determine if the expression of this gene was transcriptionally responsive to Elf5. For this purpose we focused on the cyclinD2 proximal promoter region, given the presence of a strong Elf5-binding site in this segment. The mouse cyclinD2 promoter was cloned into pGL3-basic vector and assayed for reporter activity. As a control, we generated a mutant pGL3-Ccnd2 promoter where the core GGA element of the Elf5-binding site was mutated. We utilized MCF-7 cells for the transient transfection experiments since these cells have been shown to lack endogenous Elf5 expression[5,6]. When transfected in these cells, pGL3-Ccnd2 promoter showed significant luciferase activity compared to the empty pGL3 vector suggesting that the promoter was active in these cells. Next, the reporter plasmid was cotransfected with either an expression plasmid encoding for HA-tagged Elf5, HA-tagged Elf3 (a closely related family member) or an empty HA-control vector. The expression of the HA-tagged Elf5 and HA-tagged Elf3 proteins were confirmed by western blot analysis with anti-HA antibodies (Additional File 6, Fig S5). As demonstrated in Fig. 4D, expression of both Elf3 and Elf5 resulted in decreased levels of reporter activity, although the effects of Elf5 were clearly more pronounced (4-5 fold repression). This repressive effect of Elf5 was significantly, although not completely, relieved when the pGL3-Ccnd2 promoter containing the mutant Elf5-binding site was utilized under the same conditions. This data suggests that Elf5 can act as a repressor and inhibit the activity of the proximal mouse cyclinD2 promoter.

### Expression pattern of Elf5 and cyclin D2 in mouse mammary glands

**Figure 5:**
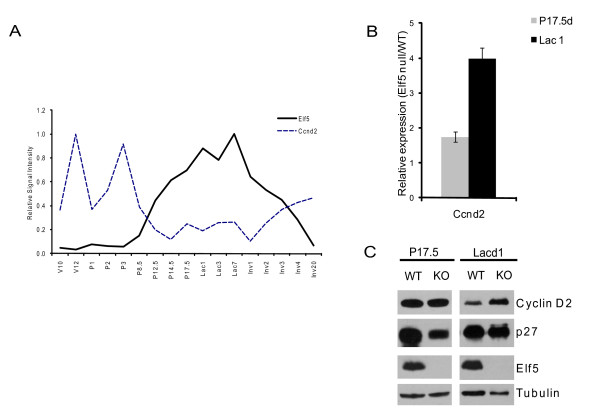
**Expression analysis of cyclin D2 as a potential Elf5 target gene**. **A**. The expression pattern of cyclin D2 and Elf5 during different stages of mammary gland development. The expression profile was generated by analysis of microarray data from Stein et al[24]. **B**. *Relative expression of cyclin D2 mRNA by real time RT-PCR during mouse mammary gland development*. Total RNA from wild type (WT) and K14-Cre/Elf5^f/f ^(null) mouse mammary glands at pregnancy day 17.5 and lactation day 1 were analyzed for relative expression of Ccnd2 mRNA levels by real time RT-PCR. The housekeeping gene Gapdh was used to normalize gene expression. Results are from two or more independent experiments. **C**. *Expression of cyclin D2 protein during mouse mammary gland development*. Protein extract from wild type (WT) and K14-Cre/Elf5^f/f ^knockout (KO) mouse mammary glands at pregnancy day 17.5 and lactation day 1 were analyzed by western blot with antibodies against cyclin D2 and p27. Equal loading was assessed by anti-β-tubulin antibodies and the absence of Elf5 in KO was confirmed by anti-Elf5 antibodies.

The expression pattern of cyclin D2 in mammary glands is quite dynamic. In one study, it was reported that cyclin D2 was not easily detectable in mammary glands at any stages of development as measured by Western blot analysis[21]. However, immunostaining data suggested that in virgin glands, cyclinD2 protein was localized predominantly in the myoepithelial cells. On the other hand, cyclinD2 was reported to be expressed in both myoepithelial and luminal epithelial cells at the RNA level in normal human breast samples[23]. Given this discrepancy, we decided to re-examine the expression profile of cyclin D2 using data from a microarray analysis that was performed on mammary glands obtained at different stages of development spanning the virgin state to involution[24]. The expression profile shown in Fig 5A clearly demonstrate a dynamic expression of cyclin D2 with a steady level throughout the virgin stages and early pregnancy but then dramatic reduction at mid pregnancy. During the same period, Elf5 showed an opposite pattern with low expression levels during virgin stages and early pregnancy and a progressive surge in expression from P12.5 onwards that coincided with the downregulation of cyclin D2. This reciprocal mode of expression is further suggestive of Elf5's role as a potential repressor keeping cyclin D2 expression in check.

If Elf5 is an important transcriptional regulator of cyclin D2, we hypothesized that in its absence; there will be changes in the expression of cyclin D2. To test this, we isolated total RNA from wild type control and Elf5-null mammary glands, generated cDNAs and performed real time PCR. While in P17.5 mammary glands, the expression of cyclin D2 was modestly higher in Elf5 knockout, by lactation day 1, this difference was significantly more pronounced (Fig 5B). To examine if these changes in transcript also correlated with protein level, we performed western blot analysis with mammary gland extracts. In agreement with mRNA levels, cyclin D2 protein was upregulated in the samples from lactation day 1 in Elf5-null mammary glands compared to wildtype control (Fig 5C). We also examined the expression of p27^kip1^, a cyclin dependent kinase inhibitor shown to be important in activation of cyclin D-CDK4 complexes[25]. Indeed, in the MMTV-cyclin D2 overexpressing mice, the mammary gland phenotype has been attributed in part to increased p27^kip1 ^protein levels[21]. However, the interactions between these cell cycle regulators are likely to be complex and context dependent. For example, cell culture based studies have shown that overexpression of cyclin D2 affect the translocation of p27 from the nucleus to the cytoplasm and promotes its degradation[26]. Western blot analysis showed that although there was a modest reduction in protein levels of p27^kip1 ^in p17.5 mammary glands in the absence of Elf5, the expression was restored to normal levels in lactating animals. The significance of this alteration in p27 levels is currently unknown and worth future investigation.

### Expression of Ccnd2 in the absence of Elf5

To determine the transcriptional effects of Elf5 on Ccnd2 in a more physiological setting, we decided to examine mammary epithelial cells (MECs) isolated from Elf5 conditional knockout (Elf5^f/f^) animals. We have previously shown[10] that in these MECs, Elf5 can be inactivated by expression of Cre recombinase by using adenoviral vectors (Ad-Cre). MECs were harvested from Elf5^f/f ^mice and infected with either Ad-Cre or Ad-GFP and cultured on basement membrane matrix for varying time points. As we have demonstrated before, infection of MECs with adenovirus expressing Cre resulted in a significant knockdown of Elf5 expression by 48 h after infection with virtually no detectable protein after 72 h. To examine cyclin D2 levels, protein lysates were harvested from MECs and western blots were performed. Loss of Elf5 leads to a strong increase in Ccnd2 further supporting the notion that Elf5 acts as a repressor of the *Ccnd2 *gene (Additional File 7, Fig S6).

**Figure 6:**
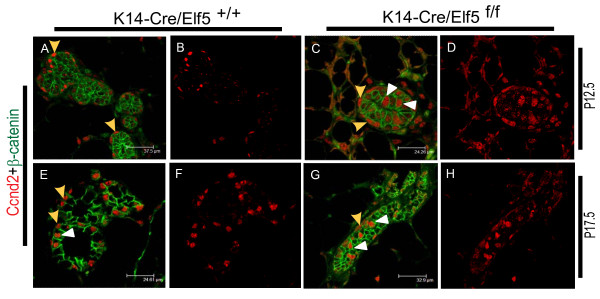
**Altered expression of Ccnd2 and p27 during pregnancy**. (A-D) P12.5 (E-H) P17.5. At P12.5, in WT tissues Ccnd2 was expressed exclusively in the basal cells (yellow arrowhead) (A). However, in Elf5-deficient tissue (K14-Cre/Elf5^f/f^) at P12.5 frequently cells were observed in luminal compartment that were positive for Ccnd2 (yellow arrowhead=basal position, white arrowhead=luminal position in C). At later time points during pregnancy (P17.5), Ccnd2 was expressed predominantly at the basal cells (yellow arrowhead) in the WT tissue (E). Few luminal cells were positive for Ccnd2 (white arrowhead). In the Elf5-deficient tissues, large number of Ccnd2 positive cells was observed in the luminal compartment (white arrowhead, G). Yellow arrowheads show localization of Ccnd2 in basal cells.

To further probe the effects of loss of Elf5 during pregnancy, we compared mammary glands from K14-Cre/Elf5^f/f ^animals and their wildtype type littermate controls (K14-Cre/Elf5^+/+^) by immunofluorescent staining with anti-Ccnd2 antibodies. At P12.5, in WT mammary glands, Ccnd2 was predominantly restricted to the outer myoepithelial cells, where they showed a patchy expression pattern whereas no staining was detected in the luminal cells. In contrast, in Elf5-null mammary glands, Ccnd2 expression clearly extended into the luminal cells (Fig 6). The altered expression profile of Ccnd2 was also observed in the P17.5 day samples, where more luminal cells stained positive for Ccnd2 in the collapsed ductal structures that are present in the Elf5-null mammary glands. These results suggest that in normal luminal cells, Elf5 keeps cyclin D2 expression in check and that in its absence, cyclin D2 is turned on in at least a subset of the luminal cells. It has been shown that in the absence of Elf5, there is decreased proliferation in the pregnant mammary glands[7,10]. Interestingly it was also shown that there is an increase in CD61+ luminal progenitors in the Elf5-null mammary epithelium[9]. We posit that the luminal cells with higher levels of cyclin D2 might represent a selective population of these luminal progenitor cells, which possess a higher proliferative potential.

## Conclusions

The identification of Ccnd2 as a direct target of Elf5 is a novel finding and has implications for the proliferative decisions of mammary epithelial cells during normal development as well as in cancer. In this context, it is also interesting to note that a closer examination of the recently generated microarray analysis of sorted mammary stem/progenitor cell populations reveal that in both the human mammary stem cell (CD49^hi ^Epcam^low ^enriched) and bipotent progenitor cell populations (Ma-CFC enriched), CCND2 is one of the most highly expressed transcript[27,28]. This is particularly important since our preliminary studies suggest that loss of Elf5 during pregnancy leads to increase in stem and progenitor cell population (unpublished data). Although in this study we demonstrate Elf5 as one transcription factor that keeps cyclin D2 repressed, this important cell cycle regulator is likely to be controlled by additional transcription factors. Indeed there is published data linking Stat5, Sp1, myc and GATA4 to the transcriptional regulation of cyclin D2 in a variety of cell types including immune cells and cardiomyocytes[29-31]. Of these, the Stat5 connection is potentially the most interesting, given the crosstalk between Stat5 and Elf5 in the prolactin mediated signaling pathway in mammary glands and hence warrants further investigation[10,32]. The availability of a set of genomic targets of Elf5 in mammary glands serves as a valuable tool for probing the molecular function of this transcription factor in mammary gland biology and has now set the stage for a rigorous global examination of the Elf5-regulated transcriptome by next generation sequencing.

## Methods

### Animals

The Elf5-deficient mice utilized in this study have been described previously[10]. Mice of mixed genetic strain background (129/Sv × C57BL/6) were used for most experiments. The genotype of the control wildtype (WT) animals were either K14-Cre/Elf5^+/+ ^or Elf5^f/f ^whereas Elf5 conditional knockouts were K14-Cre/Elf5^f/f^. Animal procedures were conducted in compliance with the guidelines of the IACUC Committee of the State University of New York at Buffalo. For assessing the pregnancy stages of mammary glands, the mice were mated and inspected for the presence of vaginal plugs in the mornings. The day of the vaginal plug was counted as day 0.5 d of pregnancy.

### Assessment of Elf5 antibody for ChIP

Commercially available anti-ELF5 antibodies were tested for ChIP by using the HEK293 UAS-TK-Luc cells [33] according to the method previously described [15]. Two million cells were cultured in 100 mm plates in DMEM containing 10% FBS and 1 mg/ml puromycin (Sigma-Aldrich). When cells reached 30% confluence, 6 μg of plasmid DNA pCMV-HA-Gal4DBD or pCMV-HA-Gal4DBD-ESE2 [13] was transfected using Fugene 6 (Roche Applied Sciences). After 48 hours, transfected and untransfected control cells were harvested for downstream application. The crosslinking, immunoprecipitation, washing, elution, reverse crosslinking and proteinase K treatment were performed according to the manufacturer's directions described in the Chromatin Immunoprecipitation (ChIP) Assay Kit (Millipore). Anti-Gal4 DBD and anti-Elf5 (N-20) antibodies were obtained from Santa Cruz Biotechnology. The immunoprecipitated DNA was purified with PCR purification kit (QIAGEN) and eluted in a final volume of 50 μl. PCR analysis of ChIPed DNA was performed with primers P1 (5'-CACACAGGAAACAGCTATGAC-3') and P2 (5'-GAATTCGCCAATGACAAGAC-3'). The PCR amplification products were analyzed by gel electrophoresis in a 1.5% agarose gel, visualized with ethidium bromide staining and pictures were taken.

### ChIP assay of mammary glands and cloning of DNA immunoprecipitated with anti-Elf5 antibodies

Mouse mammary glands (fourth and fifth inguinal) at pregnancy day 17.5 were excised in aseptic conditions, weighed and minced in PBS. The tissue fragments were crosslinked for 5 min at RT in 8 ml of PBS containing 1% formaldehyde (Sigma-Aldrich). The reaction was stopped by adding glycine (Sigma-Aldrich) to a final concentration of 125 mM and incubated for an additional 5 min. The tissue fragments were recovered by centrifugation for 10 min at 3000 × g, rinsed thrice with 8 ml of cold PBS, then resuspended in 3 ml of cold cell lysis buffer (10 mM Tris-HCl, pH 8.0, 10 mM NaCl, 3 mM MgCl_2_, 0.5% NP-40) containing 1× of Protease *Arrest *(G-Biosciences, St. Louis, MO) and transferred to a cold Dounce homogenizer. After complete homogenization of the tissue, the nuclei were pelleted at 12,000 × g for 5 min and resuspended in 200 μl of ChIP lysis buffer (Millipore) per each 100 mg of tissue, then sonicated 16 times with 15-second cycles at setting 3 (Branson Sonifier 250) to generate fragments of 0.5 kb on average. Sonicated samples were centrifugated at 12,000 ×g for 10 min and the supernatant was transferred to a new tube. An aliquot of 190 μl was diluted 10-fold in ChIP dilution buffer (EZ-ChIP kit, Millipore) and pre-cleared with 75 μl of 50% protein G-Sepharose beads slurry containing salmon sperm DNA and BSA at 4°C for 30 min. The pre-cleared chromatin was divided in 600-μl aliquots and immunoprecipitated overnight at 4°C with 10 μg of anti-Elf5 (N-20, Santa Cruz Biotechnology) or normal goat IgG (Santa Cruz Biotechnology) or no antibody as control. A 6-μl aliquot of pre-cleared chromatin was saved as input (1%). The immunoprecipitation, washing, elution, reversing crosslink and proteinase K treatment were performed according to the manufacturer's directions described in the EZ-ChIP kit.

After proteinase K treatment, the immunoprecipitated DNA was purified with the PCR purification kit (QIAGEN) and DNA was eluted in a final volume of 50 μl. After ChIP assay, the Elf5-immunoprecipiated DNA was polished by blunt ending and amplified by PCR after addition of terminal linkers as described before[16]. The amplified PCR products were purified using Glutathione Sepharose 4B beads (Amersham Biosciences) conjugated to GST-Elf5 to enrich for Elf5 binding sites. The DNA was recovered from the GST-Elf5 beads with 300 μl of elution buffer (1% SDS, 0.1 M NaHCO_3_) then phenol extracted, precipitated and resuspended in 25 μl of TE (10 mM Tris-HCl pH 8.0, 1 mM EDTA). The recovered DNA was amplified by Jumpstart Taq polymerase (Sigma-Aldrich) and the PCR products were cloned using StrataClone PCR cloning kit (Stratagene). Bacterial colonies containing the PCR products were randomly chosen for isolation of plasmid DNA. Plasmid DNAs were checked for presence of insert by restriction digestion analysis and sequencing.

### RNA extraction and Real time RT-PCR analysis

Mouse mammary glands at pregnancy day 17.5 and lactation day 1 from wild type (WT) and K14-Cre/Elf5^f/f ^animals [10] were excised in aseptic conditions. Total RNA was extracted with TRIzol reagent, according to the manufacturer's directions (Invitrogen), then purified by phenol/chloroform extraction and treated with TURBO DNAse-free kit (Ambion) to remove genomic DNA. Total RNA (2 μg) were reverse transcribed using SuperScript II reverse transcriptase (Invitrogen) according to the manufacturer's directions. Real-time PCR was performed in a volume of 25 μl containing 1× iQ SYBR Green Supermix (Bio-Rad Laboratories), 200 nM of each forward and reverse primers (sequence of gene-specific primers are listed in Table 2 (Additional File 8), and 1 μl of cDNA. PCRs were carried out at 95°C for 8.5 min followed by 40 cycles of 95°C for 15 s and 60°C for 1 min. After completion of the PCR, a melting curve program (55-95°C with a heating rate of 2°C/min) was performed to confirm that specific PCR amplification products were generated. Relative expression was determined by the 2^-ΔΔCt ^method [34] and the housekeeping gene Gapdh was used to normalize for gene expression.

### Elf5 occupancy by ChIP assay

ChIP assay in whole tissue of mouse mammary glands at pregnancy day 17.5 was performed as describe above, with 10 μg of anti-Elf5 (N-20) (Santa Cruz Biotechnology) or normal goat IgG (Santa Cruz Biotechnology) or no antibody as a control. The immunoprecipitated DNA was analyzed by PCR for Elf5 occupancy and performed in a volume of 25 μl containing 1× PCR buffer (QIAGEN), 1× Q-solution (QIAGEN), 200 μM dNTPs, 2 ng/ml of each forward and reverse primers (primer sequences are listed in Table 2), 1.25 U Taq DNA polymerase (QIAGEN) and 2 μl of template. The PCRs were carried out at 95°C for 4 min, followed by 35 cycles of 95°C for 45 s, 56°C or 58°C for 45 s and 72°C for 45 s, with a final extension step at 72°C for 5 min. The PCR amplification products were analyzed by gel electrophoresis.

### Protein extract and Western blot analysis

Mouse mammary glands (fourth and fifth inguinal) at pregnancy day 17.5 and lactation day 1 from wild type (WT) and K14-Cre/Elf5^f/f ^animals were excised in aseptic conditions and pulverized into powder in a mortar containing liquid nitrogen, then resuspended in 500 μl of RIPA buffer (50 mM Tris-HCl pH 8.0, 150 mM NaCl, 1 mM EDTA, 1% Triton X-100, 1% sodium deoxycholate, 0.1% SDS) supplemented with 1× Protease *Arrest *(G-Biosciences) and phosphatase inhibitors (1 mM Na_3_VO_4_, 10 mM NaF) and extracted for 30 min at 4°C. The samples were centrifuged at 13,000 × g for 30 min and supernatant was collected as extracted protein. Protein extract were resolved by SDS-PAGE and electrophoretically transferred to PVDF (polyvinylidene difluoride) membranes (Bio-Rad). After blocking, the membranes were probed with primary antibodies followed by a second incubation with secondary antibodies (diluted at 1:20,000 or 1:30,000) conjugated to HRP. Chemiluminescent detection of HRP-conjugated secondary antibodies was accomplished using the LumiGLO Reserve Chemiluminescent Substrate kit (KPL, Inc). The primary antibodies were anti-cyclin D2 (Santa Cruz Biotechnology) diluted at 1:4,000, anti-p27 (Santa Cruz Biotechnology) diluted at 1:4,000 and anti-Elf5 (Santa Cruz Biotechnology) diluted at 1:1,000. For loading control, the membranes were also probed with mouse anti-β tubulin (Chemicon International) diluted at 1:15,000 and HRP-conjugated antimouse IgG antibody diluted at 1:40,000.

### Bioinformatics analysis

Sequenced Elf5-ChIPed DNA clones were analyzed with the BLAST search of the mouse genome database at NCBI http://blast.ncbi.nlm.nih.gov/Blast.cgi or ENSEMBL to identify potential target genes. Gene located within 100 kb of the ChIPed DNA were considered as putative Elf5 target genes. Functional categories of the putative Elf5 target genes were based on gene ontology described in the Mouse Genome Informatics http://www.informatics.jax.org/. ChIPed DNA sequences were analyzed for conservation using the UCSC Genome Bioinformatics site http://genome.ucsc.edu/.

### Plasmid

The minimal promoter region of the mouse cyclin D2 was amplified by PCR from genomic DNA using forward primer 5' GTT ATC AGG AGT CAT AGC TTG AGG 3' and reverse primer 5' AAG GTG GGC GAG CGG AGC CTC. The amplified product was cloned into pSC-A-amp/kan (Strataclone PCR cloning Kit) and then a 645 bp fragment (-545 to +100) transferred into pGL3-basic vector using HindIII restriction enzyme. The orientation and lack of any PCR-induced mutation was confirmed by sequence analysis. The mutant pGL3-Ccnd2 promoter where the Elf5-binding site was altered (GAGG > TCTA) was generated by a two-step PCR procedure described before and verified by sequencing[35].

### Gelshift analysis and purification of GST-proteins

DNA binding reactions were performed as described previously[36]. Briefly, double-stranded oligonucleotides were labeled with [^32^P]dCTP, using Klenow fragments to fill in the overhanging 5' ends. Binding reaction mixtures were incubated at room temperature in 1× DNA binding buffer (20 mM HEPES [pH 7.9], 75 mM KCL, 2.5 mM MgCl_2_, 1 mM EDTA, 0.5 mM dithiothreitol and 10% glycerol) containing recombinant protein, 1 μg of poly(dA-dT) and the labeled probe. Reactions were then electrophoresed on a 5% nondenaturing polyacrylamide gel. Wildtype and mutant GST-Elf5 proteins were synthesized as described previously[37]. Specific mutations in the ETS-domain of Elf5 were introduced by a two-step PCR procedure described before[35].

### Cell culture, transfections, and reporter assays

MCF-7 cells were maintained in Dulbecco Modified Eagle Medium supplemented with 10% fetal bovine serum and antibiotics. Transient transfections were performed in 6-well plates using Fugene6 (Roche) in MCF-7 cells following the manufacturer's recommendations. One microgram each of CMV-HA, CMV-HAElf5, CMV-HAElf3 and luciferase reporter constructs were transfected per well along with 0.25 μg of CMVLacZ plasmid DNA to serve as an internal control for transfection efficiency. Cells were harvested 48 hours post-transfection and reporter assays were performed as described previously[35]. Expression of the HA-tagged Elf5 and Elf3 proteins was detected by western blot analysis with anti-HA antibodies (Roche, 1:5000 dilution).

### Primary mammary epithelial cell (MEC) culture and viral infection

Isolation and adenoviral transduction of primary mammary epithelial cells were performed as described previously [7]. Briefly, primary mammary epithelial cells obtained from Elf5^f/f ^animals were transduced with Adenovirus-Cre (Ad-Cre) in suspension for 45 min before they were plated on BM (Basement Membrane) matrix (Matrigel, BD Biosciences). Cells were cultured in Assay Media [DMEM/F12 medium containing Insulin (5 μg/ml), Hydrocortisone (1 μg/ml), EGF (10 ng/ml), FBS (10%), Penicilin/Streptamycin (1×), Gentamycin (50 μg/ml)].

### Immunofluorescence analysis

For immunofluorescence analysis, mammary gland specimens were fixed overnight in neutral buffered formalin [3.7% formaldehyde buffered to pH 6.8-7.2 with monobasic and dibasic sodium phosphate], dehydrated and embedded in paraffin. Tissue blocks were sectioned into 5 μm sections which were baked for 30 min at 60°C and then deparaffinized. Heat-induced antigen retrieval was performed by microwaving sections in 10 mM sodium citrate, pH 6.0 for 20 min. After blocking with M.O.M. kit (Vector Laboratories), sections were incubated with primary antibodies overnight at 4°C, followed by 45-min incubation with secondary antibodies. The slides were mounted with Vectashield (Vector Laboratories), and the immunofluorescence was viewed under Confocal Microscopy (Leica). The following antibodies and dilutions were used for immunofluorescence: Ccnd2 (Santa Cruz) at 1:200, and anti-β-catenin antibody (1:500, Sigma).

## Authors' contributions

REH and RC carried out the ChIP experiments, sequence alignments, participated in the immunostaining experiments, processed animal samples and drafted the manuscript. RR performed the gelshift experiments and performed data analysis. KS did the reporter assays and helped with animal studies. QZ, WL, MSH and MJB performed bioinformatics analysis. SS, REH and RC conceived of the study, and participated in its design and coordination and prepared the manuscript. All authors read and approved the final manuscript.

## Supplementary Material

Additional file 1 **Table 1 List of putative Elf5 target genes**. Shown are the genes that are located within 100 Kb of the isolated Elf5-ChIPed DNAs and the size of the immunoprecipitated DNA fragment that was sequenced. Click here for file

Additional file 2 **Figure S1 Relative location and the genomic context of the Elf5 ChIPed segment**. Select groups of putative Elf5 target genes chosen for further evaluation are highlighted with their genomic organization, chromosome number and the position of the ChIPed DNA fragment. Click here for file

Additional file 3 **Figure S2 Sequence conservation of the upstream Elf5-ChIPed region**. A 10 kb region 5' of the CCND2 gene was compared between different species by using the GenomeVISTA program http://genome.lbl.gov/vista/index.shtml. The segment bound by Elf5 was highly conserved among several species as indicated by the box. Click here for file

Additional file 4 **Figure S3 Sequence of the proximal CcnD2 promoter and the upstream region**. The core GGAA Elf5-binding sequence is boxed. Click here for file

Additional file 5 **Figure S4 Purified GST-Elf5 WT and the two DNA-binding deficient mutants**. GST-Elf5 wildtype and GST-Elf5 mutants were purified using GST-agarose and eluted samples were run on a SDS-PAGE gel to assess purity and amount of the proteins. The WT1 and WT2 samples represent two independently purified fractions. The mutants, MT1 and MT2 were K to A substitution at amino acid 216 and R to A substitution at amino acid 219 respectively, of the mouse Elf5 protein. Click here for file

Additional file 6 **Figure S5 Western blot demonstrating the expression of HA-Elf5 and HA-Elf3 in transient transfection experiments**. The expression of the HA-epitope tagged Ets proteins was detected by anti-HA antibodies. Click here for file

Additional file 7 **Figure S6 Increase in Ccnd2 expression in the absence of Elf5**. Elf5^f/f ^primary mammary epithelial cells (MECs) were transduced with Ad-Cre in suspension and plated on BM matrix **(A)**. Western blot analysis of protein lysates from Elf5^f/f ^MECs transduced with Ad-Cre resulted in increased Ccnd2 expression. Elf5 is absent in Ad-Cre-Elf5^f/f ^cells **(B)**. Click here for file

Additional file 8 **Table 2**. Primer sequences used for occupancy analysis of putative Elf5 target genes, real time RT-PCR and gelshift experiments. Click here for file
