# Supplementary figures and images for: Genome-wide search identifies Ccnd2 as a direct transcriptional target of Elf5 in mouse mammary gland
Source: BMC Mol Biol. 2010 Sep 10;11:68. doi: 10.1186/1471-2199-11-68 (PMC2949602; doi:10.1186/1471-2199-11-68)

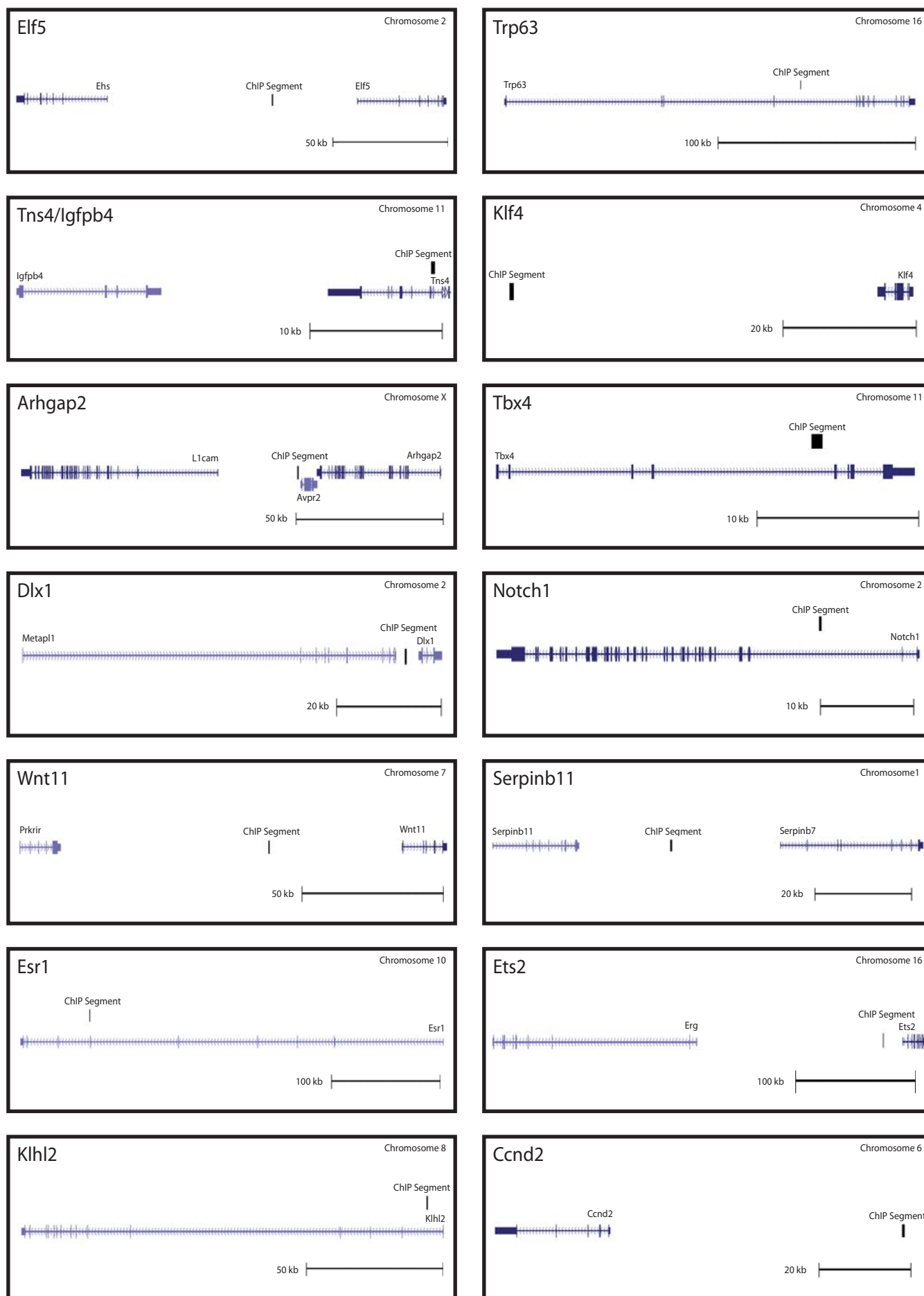

Figure S1

Supplement: Additional file 2 — Figure S1 Relative location and the genomic context of the Elf5 ChIPed segment. Select groups of putative Elf5 target genes chosen for further evaluation are highlighted with their genomic organization, chromosome number and the position of the ChIPed DNA fragment. [file 1471-2199-11-68-S2.PDF]

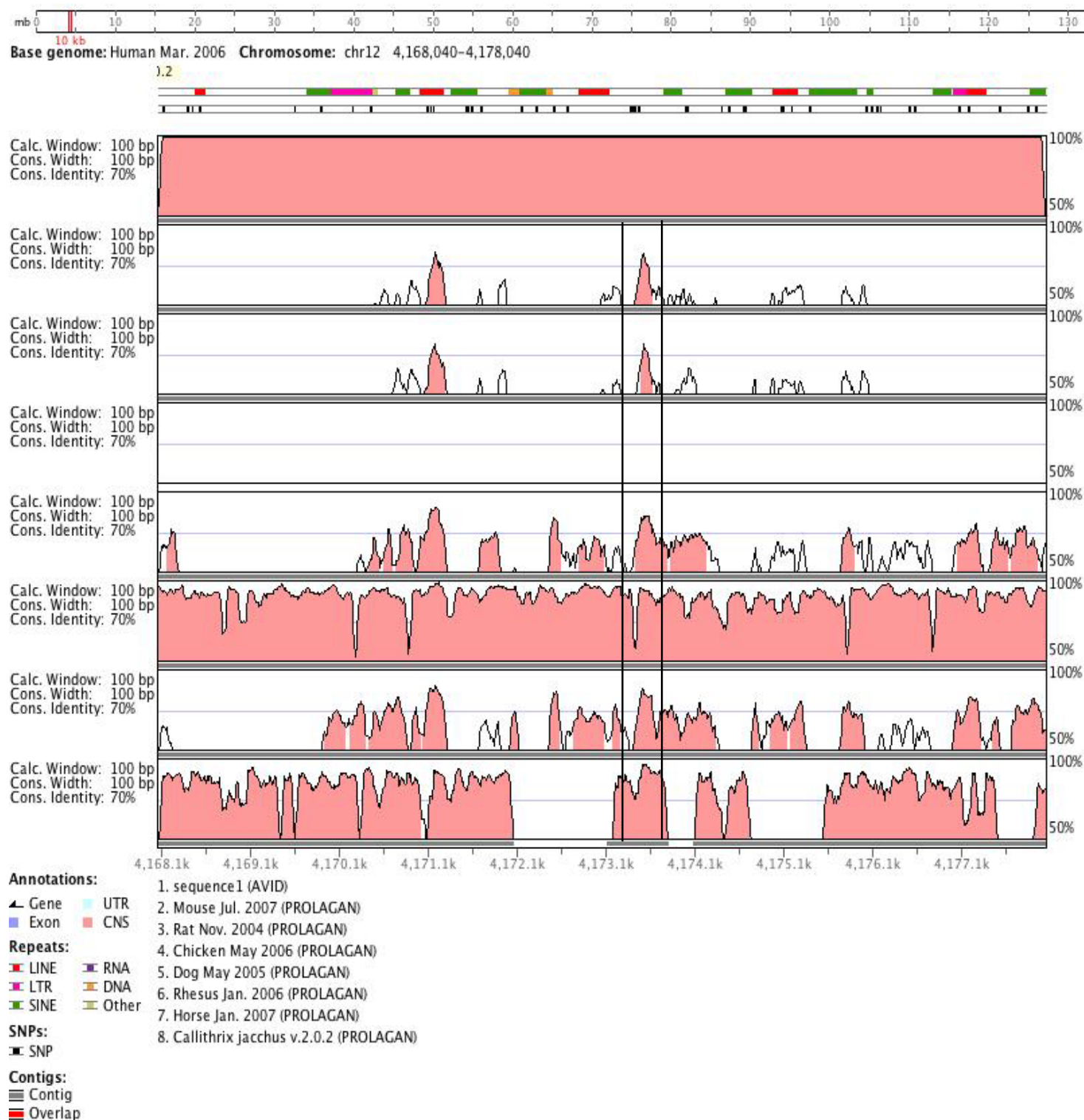

Figure S2.

Supplement: Additional file 3 — Figure S2 Sequence conservation of the upstream Elf5-ChIPed region. A 10 kb region 5' of the CCND2 gene was compared between different species by using the GenomeVISTA program http://genome.lbl.gov/vista/index.shtml. The segment bound by Elf5 was highly conserved among several species as indicated by the box. [file 1471-2199-11-68-S3.PDF]

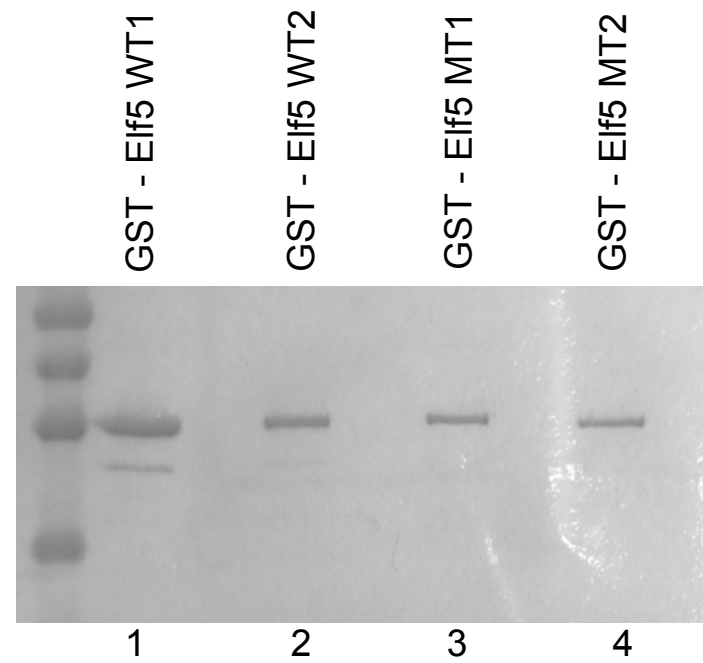

Figure S4.

Supplement: Additional file 5 — Figure S4 Purified GST-Elf5 WT and the two DNA-binding deficient mutants. GST-Elf5 wildtype and GST-Elf5 mutants were purified using GST-agarose and eluted samples were run on a SDS-PAGE gel to assess purity and amount of the proteins. The WT1 and WT2 samples represent two independently purified fractions. The mutants, MT1 and MT2 were K to A substitution at amino acid 216 and R to A substitution at amino acid 219 respectively, of the mouse Elf5 protein. [file 1471-2199-11-68-S5.PDF]

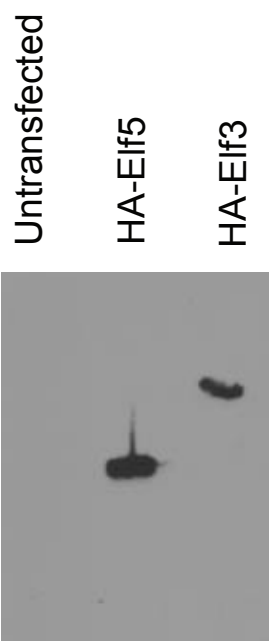

Figure S5.

Supplement: Additional file 6 — Figure S5 Western blot demonstrating the expression of HA-Elf5 and HA-Elf3 in transient transfection experiments. The expression of the HA-epitope tagged Ets proteins was detected by anti-HA antibodies. [file 1471-2199-11-68-S6.PDF]

A

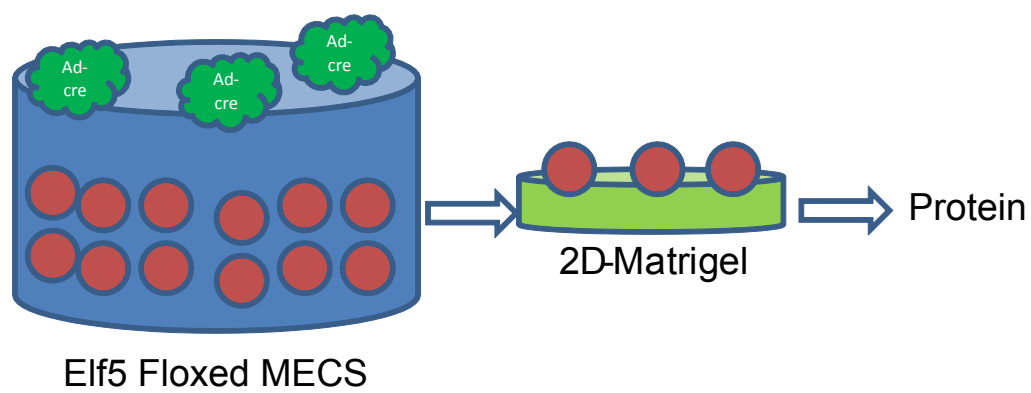

B

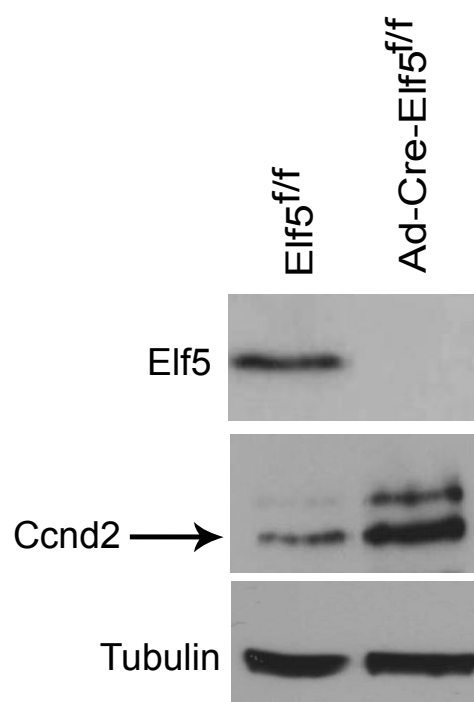

Figure S6.

Supplement: Additional file 7 — Figure S6 Increase in Ccnd2 expression in the absence of Elf5. Elf5f/f primary mammary epithelial cells (MECs) were transduced with Ad-Cre in suspension and plated on BM matrix (A). Western blot analysis of protein lysates from Elf5f/f MECs transduced with Ad-Cre resulted in increased Ccnd2 expression. Elf5 is absent in Ad-Cre-Elf5f/f cells (B). [file 1471-2199-11-68-S7.PDF]
