# Supplementary material for: Genome-wide search identifies Ccnd2 as a direct transcriptional target of Elf5 in mouse mammary gland
Source: BMC Mol Biol. 2010 Sep 10;11:68. doi: 10.1186/1471-2199-11-68 (PMC2949602; doi:10.1186/1471-2199-11-68)
Supplement: Additional file 4 — Figure S3 Sequence of the proximal CcnD2 promoter and the upstream region. The core GGAA Elf5-binding sequence is boxed. [file 1471-2199-11-68-S4.PDF]

**Mouse CyclinD2 promoter**

*HindIII*

**AAGCTT**GAGGGGCATAACCTTTATCCCTGGTTTGGCGAGGTTGCAATTTTCTCAAATAAGCCTTT -481
**TTCGAA**CTCCCCGTATTGGAAATAGGGACCAAACCGCTCCAACGTTAAAAGAGTTTATTCGGAAA

CCTTGTTCCTCATTTAGGGGTCTACTTCTAAGTTATCTGAACTCATCCACATGGTAAATCTATCTACCTGAATCTTGAAT -401
GGAACAAGGAGTAAATCCCCAGATGAAGATTCAATAGACTTGAGTAGGTGTACCATTTAGATAGATGGACTTAGAACTTA

AATGGGCTGTTTTTCTGATCACATTGCAAGCCTCCGAAGTTAGAGAGCACACACGTACACACCCTTTATGCCCCCATGGT -321
TTACCCGACAAAAAGACTAGTGTAACGTTCGGAGGCTTCAATCTCTCGTGTGTGCATGTGTGGGAAATACGGGGGTACCA

ATGCCTACAGAATGTCAGAAAGGATAATCAATAGGAATCCATGGGGTTTGTGGGTTCCCCTATCCGAGGCCCCTAGCATG -241
TACGGATGTCTTACAGTCTTTCCTATTAGTTATCCTTAGGTACCCCAAACACCCAAGGGGATAGGCTCCGGGGATCGTAC

CGGGGCTGGATGGGGAGAGGGCCTCGGAGAAGTAGGGAGAGGGGTTGGGGGTGGGGAGCGGGGATCGTGTTTGAAGTTTG -161
GCCCCGACCTACCCCTCTCCCGGAGCCTCTTCATCCCTCTCCCCAACCCCCACCCCTCGCCCCTAGCACAAACTTCAAAC

GTCAGGCCAGCTGCTGTGCTCCTTAATAACAAGAGGGAAGGGGGGGGGGAGAGGGAGGGAAAGATTGAAAGGAGGGGAGG -81
CAGTCCGGTCGACGACACGAGGAATTATTGTTCTCCCTTCCCCCCCCCCTCTCCCTCCCTTTCTAACTTTCCTCCCCTCC

GACGCTAGAGGAGGGGAGGAAAGGGGGAGGAGGAACCTGAGAGGGGGAGGAGATCTAACTGCCCTTCCAGCTTGCGTCAC -1
CTGCGATCTCCTCCCCTCCTTTCCCCCTCCTCCTTGGACTCTCCCCCTCCTCTAGATTGACGGGAAGGTCGAACGCAGTG

*Elf5-RE*

TGCCTGAGCGAGAGAGGAGAGCGAGCTGAGGAGAGCCGGGCAGTTCGGAGGGAAGGACCGGTGCGAGTCAGGCGGCCCTT
ACGGACTCGCTCTCTCCTCTCGCTCGACTCCTCTCGGCCCGTCAAGCCTCCCTTCCTGGCCACGCTCAGTCCGCCGGGAA

GAGGCTCCGCTCGCCCACCTTC
CTCCGAGGCGAGCGGGTGGAAG

**Mouse Cyclin D2 upstream element**

GGAGGCCATATCTGGCAGAGGGATGCAGGTACCAGAACCCCGCCCTACCAGCAGCAATGATGGAAATCCTGCCTCATATA
CCTCCGGTATAGACCGTCTCCCTACGTCCATGGTCTTGGGGCGGGATGGTCGTCGTTACTACCTTTAGGACGGAGTATAT

AATTCTGCTGAAAGAGAGATTGGGTGTGGGTTGGGAGCTACAGATTAGAAAGGAAAAGGATTACAGTAGTATTCAAACTA
TTAAGACGACTTTCTCTCTAACCCACACCCAACCCTCGATGTCTAATCTTTCCTTTTCCTAATGTCATCATAAGTTTGAT

*Elf5-RE*

AGTTTGAGCCCCGCCCCCCCAAATGAGTCCCCTTGACAAACTCCAGCTTAAAAACCTCCTCTGCAGAATGGAGACTACCT
TCAAACTCGGGGCGGGGGGGTTTACTCAGGGGAACTGTTTGAGGTCGAATTTTTGGAGGAGACGTCTTACCTCTGATGGA

TCCTTTCCAGGTCCCCACCAAGTGAGCTGATGTTGTGGGTGCTGAGACAAGCTCTGTGTCTAACTCAGAGTCATTACTGG
AGGAAAGGTCCAGGGGTGGTTCACTCGACTACAACACCCACGACTCTGTTCGAGACACAGATTGAGTCTCAGTAATGACC

CTTACATTACAATCCACACACTGCTGTTCTGAAGATGGCATTACTTGGGGGACAGCAGGAGACCCCCCAAAACCTGAGAC
GAATGTAATGTTAGGTGTGTGACGACAAGACTTCTACCGTAATGAACCCCCTGTCGTCCTCTGGGGGGTTTTGGACTCTG

CACTTCCTCCCCCAGCTCTGAGCCAGCAGGATGCTTGACATTCCAGCTGCTGGCCTCCACTGAGGACTGGTTTACACAGC
GTGAAGGAGGGGGTCGAGACTCGGTCGTCCTACGAACTGTAAGGTCGACGACCGGAGGTGACTCCTGACCAAATGTGTCG

*Elf5-RE*

AGATGTTATCTTCGGGTCTTTAATGGCACTCATTTAGGTTATTAACACCTTAGCTCTAATCAGATTAACCTCTGTTTGAA
TCTACAATAGAAGCCCAGAAATTACCGTGAGTAAATCCAATAATTGTGGAATCGAGATTAGTCTAATTGGAGACAAACTT

Figure S3
